# Bioactive Steroids with Methyl Ester Group in the Side Chain from a Reef Soft Coral *Sinularia brassica* Cultured in a Tank

**DOI:** 10.3390/md15090280

**Published:** 2017-09-01

**Authors:** Chiung-Yao Huang, Jui-Hsin Su, Chih-Chuang Liaw, Ping-Jyun Sung, Pei-Lun Chiang, Tsong-Long Hwang, Chang-Feng Dai, Jyh-Horng Sheu

**Affiliations:** 1 Department of Marine Biotechnology and Resources, National Sun Yat-sen University, Kaohsiung 804, Taiwan; huangcy@mail.nsysu.edu.tw (C.-Y.H.); ccliaw@mail.nsysu.edu.tw (C.-C.L.); 2 National Museum of Marine Biology & Aquarium, Pingtung 944, Taiwan; x2219@nmmba.gov.tw (J.-H.S.); pjsung@nmmba.gov.tw (P.-J.S.); 3 Institute of Marine Biotechnology, National Dong Hwa University, Pingtung 944, Taiwan; 4 Department of Biochemistry, University of Toronto, Toronto, ON M5G2R3, Canada; sharonjiang1996@hotmail.com; 5 Graduate Institute of Natural Products, College of Medicine, Chang Gung University, Taoyuan 333, Taiwan; htl@mail.cgu.edu.tw; 6 Research Center for Chinese Herbal Medicine, Research Center for Food and Cosmetic Safety, and Graduate Institute of Health Industry Technology, College of Human Ecology, Chang Gung University of Science and Technology, Taoyuan 333, Taiwan; 7 Department of Anesthesiology, Chang Gung Memorial Hospital, Taoyuan 333, Taiwan; 8 Institute of Oceanography, National Taiwan University, Taipei 112, Taiwan; corallab@ntu.edu.tw; 9 Department of Medical Research, China Medical University Hospital, China Medical University, Taichung 404, Taiwan; 10 Graduate Institute of Natural Products, Kaohsiung Medical University, Kaohsiung 807, Taiwan; 11 Frontier Center for Ocean Science and Technology, National Sun Yat-sen University, Kaohsiung 804, Taiwan

**Keywords:** soft coral, *Sinularia brassica*, steroid, cytotoxic activity, anti-inflammatory activity

## Abstract

A continuing chemical investigation of the ethyl acetate (EtOAc) extract of a reef soft coral *Sinularia brassica*, which was cultured in a tank, afforded four new steroids with methyl ester groups, sinubrasones A–D (**1**–**4**) for the first time. In particular, **1** possesses a *β*-d-xylopyranose. The structures of the new compounds were elucidated on the basis of spectroscopic analyses. The cytotoxicities of compounds **1**–**4** against the proliferation of a limited panel of cancer cell lines were assayed. The anti-inflammatory activities of these new compounds **1**–**4** were also evaluated by measuring their ability to suppress superoxide anion generation and elastase release in *N*-formyl-methionyl-leucyl-phenylalanine/cytochalasin B (fMLP/CB)-induced human neutrophils. Compounds **2** and **3** were shown to exhibit significant cytotoxicity, and compounds **3** and **4** were also found to display attracting anti-inflammatory activities.

## 1. Introduction

**Figure 1:**
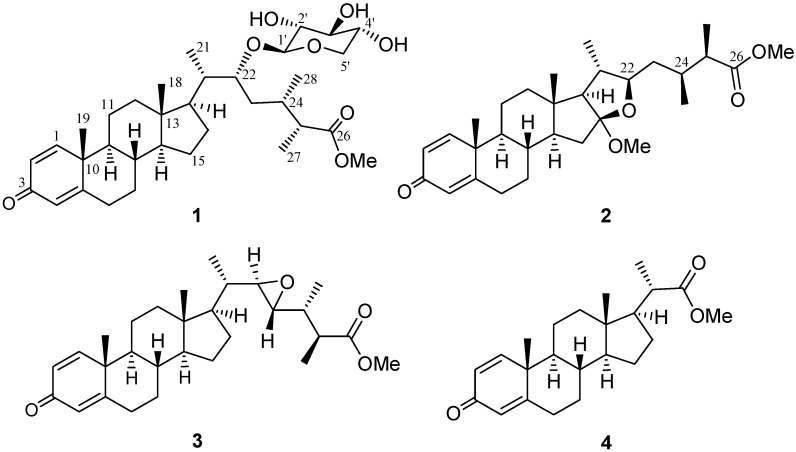
Structures of compounds **1**–**4**.

Soft corals of the genus *Sinularia* have yielded series of natural products of differing chemical types [1]. Many of these compounds have been shown to exhibit interesting bioactivities, such as cytotoxic [2,3,4,5,6,7,8], antifouling [9], and anti-inflammatory [10,11,12,13,14,15,16,17,18] activities. Previous chemical investigation of the wild-type soft coral *Sinularia brassica* (May 1898) has led to the isolation of only two new steroids from this marine organism [19]. In contrast, during the course of our investigation of the bioactive substances obtained from this reef soft coral which was later cultured in a tank, 12 new withanolide-based steroidal metabolites, sinubrasolides A–L, were discovered [20,21]. As most withanolides were isolated from terrestrial plants, and have attracted considerable attention due to their versatile bioactivities [22,23], marine withanolides discovered from reef soft corals *Minabea* sp. [24] and *Paraminabea acronocephala* [25], as well as the cultured *S. brassica*, have also been found to possess unusual withanolide-based structures and/or exhibit interesting biological activities, we further continued our exhaustive investigation of the cultured *S. brassica* with the aim of discovering new bioactive metabolites for further biomedical application. This study led to the discovery of four new non-withanolidal steroids, sinubrasones A–D (**1**–**4**) (Figure 1). The structure of **1** is unusual as it possesses a *β*-d-xylopyranose at C-22 of the side-chain. The structures of **1**–**4** were established by extensive spectroscopic analyses, including 2D nuclear magnetic resonance (NMR) spectroscopy. The in vitro cytotoxicities of **1**–**4** against four cancer cell lines, murine macrophage-like (P388D1), human T-lymphoid (MOLT-4), human erythroleukemia (K-562), and human colon carcinoma (HT-29), were measured. The abilities of new compounds **1**–**4** to inhibit superoxide anion generation and elastase release in *N*-formyl-methionyl-leucyl-phenylalanine/cytochalasin B (fMLP/CB)-induced neutrophils were also assayed. Herein, we report the isolation, structure elucidation and biological activities of these compounds.

## 2. Results and Discussion

A reef soft coral *S. brassica* further cultured in a tank was collected by hand from a cultivation pool of at the National Museum of Marine Biology and Aquarium, Taiwan, in January 2010. The organisms were further stored in a freezer until extraction. The frozen bodies were minced and extracted exhaustively with CH_2_Cl_2_ and MeOH (0.5 L × 6), as previously described [20]. Fractions 12 and 18, which contained terpenoids, as revealed by ^1^H-NMR spectra, were further purified by column chromatography and reversed-phase high-performance liquid chromatography (HPLC) to afford **1**–**4** (see Section 3).

**Figure 2:**
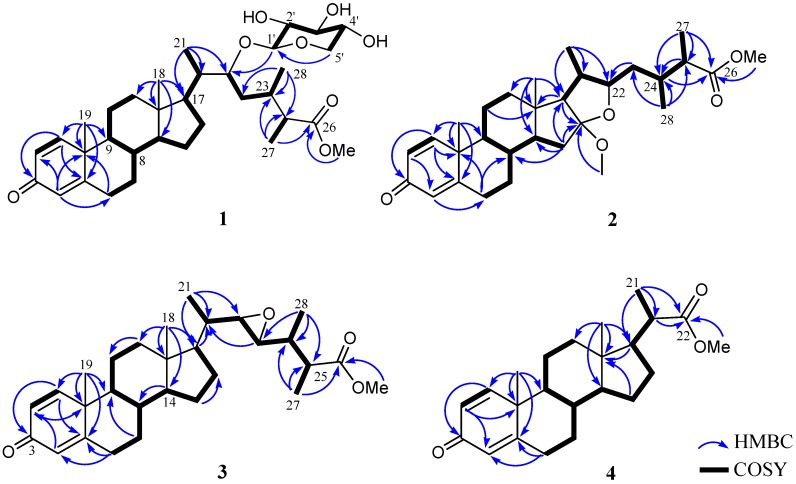
Selected correlation spectroscopy (COSY) and heteronuclear multiple bond correlations (HMBC) of **1**–**4**.

Sinubrasone A (**1**) was isolated as an amorphous solid. The high-resolution electrospray ionization mass spectrometry (HRESIMS) spectrum of **1** exhibited a [M + Na]^+^ peak at *m*/*z* 611.35545, indicating the molecular formula C_34_H_52_O_8_, requiring nine degrees of unsaturation. The infrared (IR) spectrum revealed the presence of a hydroxy group (3396 cm^−1^), an ester (1735 cm^−1^) group, and a conjugated enone (1664 cm^−1^). The ^13^C NMR spectroscopic data of **1** exhibited 34 carbon signals (Table 1), which were assigned by the assistance of a distortionless enhancement by polarization transfer (DEPT) spectra to six methyls (including a methoxy), eight sp^3^ methylenes, 12 sp^3^ methines (including five oxymethines), three sp^2^ methines, three sp^2^, and two sp^3^ non-protonated carbons (including an ester carbonyl and a ketone). The above data accounted for four of the nine degrees of unsaturation, indicating a pentacyclic structure for **1**. Proton signals (Table 1) resonating at *δ*_H_ 7.05 (1H, d, *J* = 10.0 Hz), 6.23 (1H, d, *J* = 10.0 Hz), and 6.07 (1H, s), as well as carbon signals appearing at *δ*_C_ 186.5 (C), 169.3 (C), 156.0 (CH), 127.5 (CH), and 123.8 (CH), indicated the presence of a 1,4-dien-3-one structural unit in ring A of the steroids [25,26]. The molecular framework of **1** was further established by correlation spectroscopy (COSY) and heteronuclear multiple bond correlation (HMBC) correlations (Figure 2). By comparison of the NMR data of **1** with those of cladophenol glycoside A [12], it was found that the presence of an anomeric proton at *δ*_H_ 4.30 (1H, d, *J* = 7.0 Hz) to arise from a *β*-xylopyranose moiety prossessing carbon signals at *δ*_C_ 104.5 (CH, C-1′), 73.4 (CH, C-2′), 75.9 (CH, C-3′), 69.5 (CH, C-4′), and 64.9 (CH, C-5′) (Table 1), as confirmed by HMBC correlation from H-5′ to C-1′ and the NOE correlations of H-1′/H-3′ and H-2′/H-4′. Moreover, the *β*-xylopyranose residue attached at C-22 was assigned according to an HMBC correlation from the anomeric proton (H-1′) to C-22. Thus, the planar structure of **1** was established.

**Figure 3:**
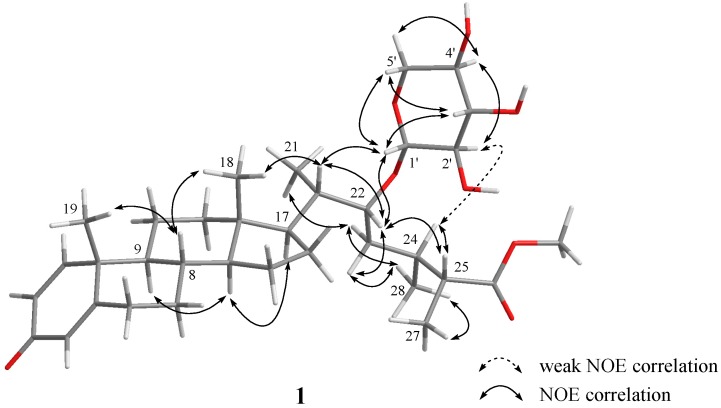
Selected nuclear Overhauser effect (NOE) correlations for **1**.

The configuration of **1** was further confirmed by analysis of their nuclear Overhauser effect (NOE) correlations. In the nuclear Overhauser enhancement spectroscopy (NOESY) spectrum of **1**, NOE correlations of H-20 with H_3_-18 and H-22, but H-22 not with H_3_-21, revealed the *β*-orientation of H-22. Moreover, correlations of H-25 with H-22 and H-24, and H_3_-27 with H_3_-28, revealed the 20*S*, 22*R*, 24*S*, 25*R* configuration of **1**. Finally, the anomeric proton (H-1′) was found to show NOE correlations with H-20 and H-22 as shown **1** in Figure 3, and H-2′ was also found to show weak NOE correlations with H-24, but H_2_-5′ did not show NOE correlation with H-24, revealed the *β*-d-xylopyranose residue of the cholesterol. On the basis of the above findings and other detailed NOE correlations, the structure of **1** was established to be that of formula **1**.

Sinubrasone B (**2**) had the molecular formula C_30_H_44_O_5_ as determined by HRESIMS and from ^13^C-NMR data. Thus, nine degrees of unsaturation were determined for **2**. The IR absorption band at 1735 and 1662 cm^−1^ indicated the presence of an ester carbonyl group and a conjugated enone, which was further supported by NMR signals resonating at *δ*_C_ 176.8, C in **2**. The ^1^H and ^13^C-NMR spectra (Table 2) displayed resonances for a carbonyl (*δ*_C_ 186.3, C), three sp^2^ methine double bonds (*δ*_H_ 7.04, 1H, d, *J* = 10.0 Hz, *δ*_C_ 155.7; *δ*_H_ 6.23, 1H, d, *J* = 10.0 Hz, *δ*_C_ 127.6; *δ*_H_ 6.07, 1H, s, *δ*_C_ 123.9), an sp^2^ non-protonated carbon (*δ*_C_ 168.9), an oxygenated non-protonated carbon (*δ*_C_ 117.9), two methoxy groups (*δ*_C_ 51.4, *δ*_H_ 3.67, s; and *δ*_C_ 49.3, *δ*_H_ 3.20, s), and an oxymethine (*δ*_C_ 86.9, *δ*_H_ 3.69, m). The remaining five degrees of unsaturation identified **2** as a pentacyclic triterpane. Proton signals (Table 2) resonating at *δ*_H_ 7.04 (1H, d, *J* = 10.0 Hz), 6.23 (1H, d, *J* = 10.0 Hz), and 6.07 (1H, s), as well as carbon signals appearing at *δ*_C_ 186.3 (C), 155.7 (CH), 127.6 (CH), 123.9 (CH), and 168.9 (C), indicated the presence of a 1,4-dien-3-one structural unit in ring A of the steroids [25,26]. Analysis of COSY correlations (Figure 2) of **2** indicated three consecutive proton sequences. The connection of the three partial structures was subsequently resolved by HMBC. Methoxyl and methyl ester groups were assigned at C-16 and C-25, respectively, based on HMBC correlations from the methoxyl proton (*δ*_H_ 3.20, s) to C-16, and from H_3_-27 to C-24, C-25, and C-26. In consideration of the degrees of unsaturation and molecular formula, an ether linkage was placed between C-16 and C-22, which further confirmed the presence of a tetrahydrofuran (THF) ring fused to ring D (Figure 2). Accordingly, the planar structure of **2** was established.

The configuration of **2** was further confirmed by NOE correlations (Figure 3). It was found that H_3_-18 (*δ*_H_ 0.83, s) showed NOE interactions with H-20 (*δ*_H_ 1.74, m) and one of the methylene protons at C-23 (*δ*_H_ 1.48, m); therefore, assuming the β-orientation of H_3_-18, H-20 and the above H-23 should also be positioned on the β-face, while the other (*δ*_H_ 1.53, m) was assigned as H-23*α*. Furthermore, H-22 was found to show NOE correlations with H_3_-21 (*δ*_H_ 1.02, d, *J* = 7.0 Hz), H-23*α* and H-24 (*δ*_H_ 2.07, dddq, *J* = 6.5, 6.5, 6.5, 6.5 Hz), and H_3_-27 showed correlation with H_3_-28 (*δ*_H_ 0.93, d, *J* = 6.5 Hz), reflecting the α-orientations of H-22, H-24 and H-25, and the 25*R* configuration of **2**. On the basis of the above findings and other observed correlations (Figure 3), the structure of sinubrasone B (**2**) was suggested.

The molecular formula of sinubrasone C (**3**) was found to be C_29_H_42_O_4_, as deduced from HRESIMS and ^13^C-NMR data, appropriate for nine degrees of unsaturation. The IR spectrum of **3** showed the presence of carbonyl groups of an ester and a conjugated enone (ν_max_ 1736 and 1662 cm^−1^), and the ^13^C-NMR (Table 2) and DEPT spectra also showed a signal of an ester carbonyl group at *δ*_C_ 175.6, C. Inspection of the ^1^H and ^13^C-NMR data of **3** suggested the presence of a 1,2-disubstituted epoxide (*δ*_C_ 63.9, CH; 59.1, CH; *δ*_H_ 2.59, dd, *J* = 4.8, 2.4 Hz; 2.52, dd, *J* = 7.6, 2.4 Hz). These data suggested that **3** possessed a 22,23-epoxide, which was corroborated by the HMBC correlations from H_3_-21 to C-22, and H_3_-28 to C-23, as well as the COSY correlation between H-22 and H-23. Additionally, H_3_-27 and H_3_-OMe displayed HMBC correlation to carbonyl carbon C-26 (Figure 2).

**Figure 4:**
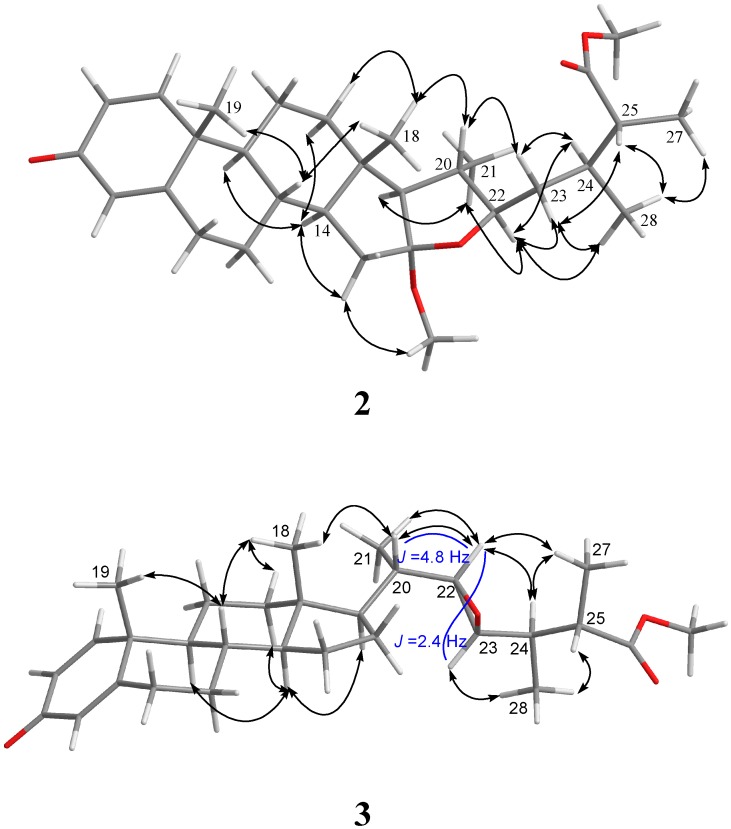
Selected NOE correlations for **2** and **3**.

The stereochemistry of **3** was determined on the basis of NOE correlations and by comparison of NMR spectroscopic data. A small coupling constant (2.4 Hz) between H-22 and H-23 suggested a *trans* conformation for both protons [27]. The NOE interactions of H_3_-18 with H-20, H-17 with H_3_-21, H-22 with H-20 and H-24, and H-23 with H_3_-28, revealed the *β*-orientations of H-22 and H-24. Further, the 22*R* and 23*R* configurations, as opposed to 22*S* and 23*S*, were confirmed by comparing the *δ* values of H-20 (*δ*_H_ 1.32), H_3_-21 (*δ*_H_ 1.00), H-22 (*δ*_H_ 2.59) and H-23 (*δ*_H_ 2.46) of known compound (22*R*,23*R*,24*R*)-3*β*-acetoxy-24-methyl-22,23-epoxy-5*α*-holestan-6-one [28] with the corresponding H-20 (*δ*_H_ 1.30), H_3_-21 (*δ*_H_ 0.99), H-22 (*δ*_H_ 2.59) and H-23 (*δ*_H_ 2.52) of **3**, while (22*S*,23*S*,24*R*)-3*β*-acetoxy-24-methyl-22,23-epoxy-5*α*-cholestan-6-one [28] with 22*S* and 23*S* configurations showed corresponding NMR signals for H-20 (*δ*_H_ 1.17), H_3_-21 (*δ*_H_ 1.09), H-22 (*δ*_H_ 2.39) and H-23 (*δ*_H_ 2.67). In addition, the NOESY spectrum of **3** showed NOE correlations between H_3_-28 with H-25, and H-24 with H_3_-27, but not between H_3_-28 and H_3_-27, revealing the 24*R* and 25*S* configurations of **3** (Figure 4). Thus, the absolute configuration of **3** was determined.

Sinubrasone D (**4**) had the molecular formula of C_23_H_32_O_3_, as determined by HRESIMS. It was also found to possess the same A–D rings as compounds **1**–**3** by comparison of NMR spectroscopic data (Table 1 and Table 2). The gross structure of **4** was determined by detailed analysis of COSY and HMBC correlations (Figure 2). The HMBC experiment of **4** further revealed the connectivity from H_3_-21 (*δ*_H_ 1.18, d, *J* = 7.0 Hz) and the methoxyl (*δ*_H_ 3.65, s) to the carbonyl carbon (*δ*_C_ 177.1). The relative configurations at C-8, C-9, C-10, C-13, C-14, C-17, and C-20 in **4** were found to be the same as those of compounds **1**–**3** by comparison of NMR data and NOE correlations.

As previous studies revealed that steroids from soft corals might possess attracting biological activities [29,30,31,32,33], we further evaluated the biological activities of these isolated steroids. Compounds **1**–**4** were evaluated in terms of their cytotoxic activities against P388D1, MOLT-4, K-562, and HT-29 cell lines using the Alamar Blue assay. Compounds **2** and **3** were found to show significant cytotoxicity against all cell lines. Compounds **1** and **4** exhibited only weak cytotoxic activity against P388D1, MOLT-4, K-562, and HT-29 cell lines (Table 3).

The anti-inflammatory activities of new compounds **1**–**4** on neutrophil pro-inflammatory responses were evaluated by measuring their ability to suppress fMLP/CB-induced superoxide anion (O_2_^−•^) generation and elastase release in human neutrophils, and the results are shown in Table 4. From the results, **4** showed significant inhibitory effect (53.6 ± 1.8%) against superoxide anion generation at 10 μM. Compounds **3** and **4** also exhibited inhibitory activities against elastase release, with the inhibition rate of 58.8 ± 4.0 and 66.3 ± 6.0% in the fMLP/CB-stimulated cells at the same concentration. The IC_50_ values of compound **4** for the superoxide anion generation and compounds **3** and **4** for inhibition of elastase release were also measured and were found to be lower than 10 μM.

## 3. Experimental Section

### 3.1. General Experimental Procedures

Optical rotations of the isolates were measured on a JASCO P1020 digital polarimeter (JASCO Corporation, Tokyo, Japan) and on a Horiba High Sensitivity Polarimeter SEPA-300 (Horiba Ltd., Kyoto, Japan). Ultraviolet spectra were recorded on a JASCO V-650 spectrophotometer (JASCO Corporation). IR spectra were recorded on a JASCO FT/IR-4100 infrared spectrophotometer (JASCO Corporation). NMR spectra were recorded on a Varian 400MR FT-NMR or Varian Unity INOVA500 FT-NMR (Varian Inc., Palo Alto, CA, USA) instrument at 400 MHz (or 500 MHz) for ^1^H and 100 MHz (or 125 MHz) for ^13^C in CDCl_3_, and the chemical shifts were referenced to residual signals of TMS (*δ*_H_ 0.00 ppm) and the CDCl_3_ (*δ*_C_ 77.0 ppm). ESIMS and HRESIMS data were obtained with a Bruker APEX II mass spectrometer (Bruker, Bremen, Germany). Silica gel (230–400 mesh, Merck, Darmstadt, Germany) was used for column chromatography. Pre-coated silica gel plates (Merck, Kieselgel 60 F-254, 0.2 mm) were used for analytical TLC. High-performance liquid chromatography was performed on a Hitachi L-2455 HPLC apparatus (Hitachi Ltd., Tokyo, Japan) with a Supelco C18 column (250 × 21.2 mm, 5 μm).

### 3.2. Animal Material

The soft coral *Sinularia brassica* used in this study was originally collected from a reef and cultured in an 80-ton cultivation tank (height 1.6 m) located in the National Museum of Marine Biology and Aquarium, Taiwan, for five years. The soft coral organisms were collected in January 2010 and were stored in a −20 °C freezer until extraction, while the voucher specimen (specimen no. 201001C1) was deposited in the Department of Marine Biotechnology and Resources, National Sun Yat-sen University. The soft coral was identified by one of the authors (C.-F.D.).

### 3.3. Extraction and Isolation

The frozen bodies of *S. brassica* (0.4 kg, wet weight) were minced and extracted exhaustively with the 1:1 mixture of CH_2_Cl_2_ and MeOH (0.5 L × 6). The combined extract was evaporated under reduced pressure and the residue was partitioned between EtOAc and H_2_O to give the EtOAc-soluble fraction. The EtOAc extract (3.7 g) was subjected to separation using a silica gel column with a gradient of EtOAc and *n*-hexane in an increasing polarity (0–100%, stepwise), and then with MeOH in EtOAc (5–50%, stepwise) to yield 24 fractions. Fraction 12 eluting with EtOAc–*n*-hexane (1:9) was further purified with acetone–*n*-hexane (1:7) to give five subfractions (12A–12F). Subfraction 12B was further separated by reversed-phase HPLC using MeOH–H_2_O (4:1) to yield **2** (0.8 mg), **3** (1.1 mg), and **4** (0.9 mg). Fraction 18 eluting with EtOAc–*n*-hexane (1:3) was separated by silica gel column chromatography with acetone–*n*-hexane (1:3) to afford four subfractions (18A–18D). Subfraction 18C was further purified by reversed-phase HPLC using MeOH–H_2_O (2:1) to yield **1** (1.3 mg).

Sinubrasone A (**1**): amorphous solid; ${\left[\alpha \right]}_{D}^{23}$ −30 (*c* 0.50, CHCl_3_); UV (MeOH) λ_max_ (log *ε*) 243 (4.2); IR (neat) ν_max_ 3396, 2941, 1735 and 1664 cm^−1^; ^13^C and ^1^H-NMR data, see Table 1; ESIMS *m*/*z* 611 [M + Na]^+^; HRESIMS *m*/*z* 611.35545 [M + Na]^+^ (calcd. for C_34_H_52_O_8_Na, 611.35544).

Sinubrasone B (**2**): amorphous solid; ${\left[\alpha \right]}_{D}^{23}$ −128 (*c* 0.25, CHCl_3_); UV (MeOH) λ_max_ (log *ε*) 244 (4.3); IR (neat) ν_max_ 2930, 1735 and 1662 cm^−1^; ^13^C and ^1^H-NMR data, see Table 2; ESIMS *m*/*z* 507 [M + Na]^+^; HRESIMS *m*/*z* 507.3089 [M + Na]^+^ (calcd. for C_30_H_44_O_5_Na, 507.3086).

Sinubrasone C (**3**): amorphous solid; ${\left[\alpha \right]}_{D}^{23}$ −68 (*c* 0.25, CHCl_3_); UV (MeOH) λ_max_ (log *ε*) 245 (4.2); IR (neat) ν_max_ 2936, 2856, 1736 and 1662 cm^−1^; ^13^C and ^1^H-NMR data, see Table 2; ESIMS *m*/*z* 477 [M + Na]^+^; HRESIMS *m*/*z* 477.2978 [M + Na]^+^ (calcd. for C_29_H_42_O_4_Na, 477.2981).

Sinubrasone D (**4**): amorphous solid; ${\left[\alpha \right]}_{D}^{24}$ −65 (*c* 0.035, CHCl_3_); UV (MeOH) λ_max_ (log *ε*) 244 (4.0); IR (neat) ν_max_ 2928, 2853, 1735 and 1662 cm^−1^; ^13^C and ^1^H-NMR data, see Table 2; ESIMS *m*/*z* 379 [M + Na]^+^; HRESIMS *m*/*z* 379.2250 [M + Na]^+^ (calcd. for C_23_H_32_O_3_Na, 379.2249).

### 3.4. Cytotoxicity Assay

Cytotoxicity assays were performed as previous reported, using an Alamar Blue assay [34,35]. Cell lines (P388D1, MOLT-4, K-562, and HT-29) were purchased from the American Type Culture Collection (ATCC). Cancer cells were plated onto 96-well microtiter plates possessing clear flat bottoms (Thermo Scientific Nunc MicroWell plate) with densities of 5 × 10^3^ to 1 × 10^4^ cells per well and incubated in a humidified 5% CO_2_ atmosphere at 37 °C. After 15 h of culture, the solutions of compounds in DMSO were added. After 72 h, attached cells were incubated with Alamar Blue (10 μL/well, 4 h). The absorbance at 595 nm was recorded using the ELISA reader. The IC_50_ values represented the concentrations of the compounds tested that could reduce cell growth by 50% under the experimental conditions.

### 3.5. Human Neutrophil Superoxide Anion Generation and Elastase Release

The human neutrophils were isolated through dextran sedimentation and Ficoll centrifugation. As previously described procedures, the assay of superoxide anion generation was measured from the SOD-inhibitable reduction of ferricytochrome C. The elastase release experiment was performed according to MeO–Suc–Ala–Ala–Pro–Val–*p*-nitroanilide as the enzyme substrate [36,37].

## 4. Conclusions

Our continuing investigations demonstrated that the cultured soft coral *Sinularia brassica* is a good source of bioactive withanolides and non-withanolidal steroids with methyl ester groups. Moreover, it is worthwhile to note here that **1**–**4** were found to be the novel steroids with a methyl ester group, and **1** with a *β*-d-xylopyranose on the side chain is quite rare. Metabolites **2** and **3** possessing a methyl ester at C-25 were shown to exhibit significant cytotoxic activities against P388D1, MOLT-4, K-562, and HT-29 cancer cell lines. Compounds **3** and **4** also exhibited notable anti-inflammatory activities in inhibition of elastase release in fMLP/CB-induced human neutrophils, and **4** could inhibit the generation of superoxide anion, too. Owing to these attractive biological activities, **2**–**4** might be useful lead compounds for future drug discoveries.

## Figures and Tables

**Table 1:** ^1^H and ^13^C-NMR spectroscopic data of **1**.

	1				
Position	*δ*_C_ *^a^* (Mult.) *^b^*	*δ*_H_ *^c^* (*J* in Hz)	Position	*δ*_C_ (Mult.)	*δ*_H_ (*J* in Hz)
1	156.0, CH	7.05 d (10.0)	1′	104.5, CH	4.30 d (7.0)
2	127.5, CH	6.23 d (10.0)	2′	73.4, CH	3.42 dd (8.5, 7.0)
3	186.5, C		3′	75.9, CH	3.52 dd (8.5, 8.5)
4	123.8, CH	6.07 s	4′	69.5, CH	3.75 ddd (9.5, 8.5, 5.0)
5	169.3, C		5′	64.9, CH_2_	4.01 dd (12.0, 5.0);
6	32.8, CH_2_	2.47 ddd (12.5, 12.5, 4.5)			3.30 d (12.0, 9.5)
		2.36 br d (12.5)	26-OMe	52.1, CH_3_	3.71 s
7	33.6, CH_2_	1.95 m; 1.04 m			
8	35.5, CH	1.63 m			
9	52.3, CH	1.04 m			
10	43.6, C				
11	22.8, CH_2_	1.70 m			
12	39.4, CH_2_	2.04 m; 1.16 m			
13	43.0, C				
14	55.0, CH	0.99 m			
15	24.5, CH_2_	1.66 m; 1.20 m			
16	27.5, CH_2_	1.68 m; 1.34 m			
17	52.6, CH	1.06 m			
18	11.9, CH_3_	0.76 s			
19	18.7, CH_3_	1.23 s			
20	39.5, CH	2.04 m			
21	12.6, CH_3_	0.92 d (6.5)			
22	81.5, CH	3.63 br d (11.0)			
23	32.3, CH_2_	1.43 ddd (14.5, 11.0, 3.5);			
		1.28 m			
24	31.5, CH	2.29 m			
25	40.9, CH	2.62 qd (6.0, 4.0)			
26	177.7, C				
27	9.9, CH_3_	1.02 d (7.0)			
28	16.3, CH_3_	0.85 d (7.0)			

*^a^* Spectrum recorded at 100 MHz in CDCl_3_. *^b^* Attached protons were deduced by distortionless enhancement by polarization transfer (DEPT) experiment. *^c^* Spectrum recorded at 500 MHz in CDCl_3_.

**Table 2:** ^1^H and ^13^C-NMR spectroscopic data of **2**–**4**.

	2		3		4	
Position	*δ*_C_ *^a^* (Mult.) *^b^*	*δ*_H_ *^c^* (*J* in Hz)	*δ*_C_ *^d^* (Mult.)	*δ*_H_ *^e^* (*J* in Hz)	*δ*_C_ *^a^* (Mult.)	*δ*_H_ *^c^* (*J* in Hz)
1	155.7, CH	7.04 d (10.0)	156.0, CH	7.05 d (10.4)	155.8, CH	7.05 d (10.0)
2	127.6, CH	6.23 d (10.0)	127.5, CH	6.23 d (10.4)	127.5, CH	6.23 d (10.0)
3	186.3, C		186.5, C		186.4, C	
4	123.9, CH	6.07 s	123.8, CH	6.07 s	123.9, CH	6.07 s
5	168.9, C		169.4, C		169.2, C	
6	32.7, CH_2_	2.46 ddd (12.0, 12.0, 4.0)	32.9, CH_2_	2.47 m	32.8, CH_2_	2.46 ddd (13.0, 13.0, 4.0)
		2.36 br d (12.0)		2.37 m		2.36 m
7	33.5, CH_2_	1.90 m; 1.07 m	33.6, CH_2_	1.96 m; 1.05 m	33.5, CH_2_	1.94 m; 1.05 m
8	35.1, CH	1.76 m	35.5, CH	1.60 m	35.5, CH	1.64 m
9	52.2, CH	1.09 m	52.4, CH	1.06 m	52.2, CH	1.08 m
10	43.6, C		43.6, C		43.5, C	
11	22.4, CH_2_	1.69 m	22.8, CH_2_	1.66 m	22.8, CH_2_	1.71 m
12	38.8, CH_2_	1.71 m	39.3, CH_2_	1.99 m	39.2, CH_2_	1.97 ddd (13.0, 3.0, 3.0)
		1.21 m		1.21 m		1.28 m
13	40.9, C		43.0, C		42.7, C	
14	54.6, CH	1.36 dd (12.0, 5.5)	55.0, CH	1.02 m	55.0, CH	1.08 m
15	33.5, CH_2_	1.96 dd (12.0, 5.5)	24.6, CH_2_	1.63 m	24.4, CH_2_	1.62 m
		1.31 dd (12.0, 12.0)		1.17 m		1.19 m
16	117.9, C		26.9, CH_2_	1.93 m; 1.60 m	27.0, CH_2_	1.70 m; 1.30 m
17	70.9, CH	1.65 m	55.8, CH	1.30 m	52.7, CH	1.60 m
18	15.3, CH_3_	0.83 s	12.2, CH_3_	0.73 s	12.2, CH_3_	0.76 s
19	19.2, CH_3_	1.24 s	18.7, CH_3_	1.23 s	18.7, CH_3_	1.23 s
20	38.1, CH	1.74 m	38.5, CH	1.30 m	42.4, CH	2.43 m
21	18.8, CH_3_	1.02 d (7.0)	15.9, CH_3_	0.99 d (7.2)	17.0, CH_3_	1.18 d (7.0)
22	86.9, CH	3.69 m	63.9, CH	2.59 dd (4.8, 2.4)	177.1, C	
23	38.5, CH_2_	1.53 m; 1.48 m	59.1, CH	2.52 dd (7.6, 2.4)		
24	33.3, CH	2.07 dddq (6.5, 6.5, 6.5, 6.5)	39.2, CH	1.54 m		
25	43.6, CH	2.49 dq (6.5, 6.5)	42.9, CH	2.48 m		
26	176.8, C		175.6, C			
27	11.9, CH_3_	1.08 d (6.5)	14.5, CH_3_	1.21 d (7.2)		
28	16.3, CH_3_	0.93 d (6.5)	14.8, CH_3_	1.02 d (7.2)		
16-OMe	49.3, CH_3_	3.20 s				
22-OMe					51.4, CH_3_	3.65 s
26-OMe	51.4, CH_3_	3.67 s	51.5, CH_3_	3.69 s		

*^a^* Spectrum recorded at 125 MHz in CDCl_3_. *^b^* Attached protons were deduced by DEPT experiment. *^c^* Spectrum recorded at 500 MHz in CDCl_3_. *^d^* Spectrum recorded at 100 MHz in CDCl_3_. *^e^* Spectrum recorded at 400 MHz in CDCl_3_.

**Table 3:** Cytotoxicity (IC_50_ μM) of compounds **1**–**4**.

	Cell lines IC_50_ (μM)			
Compound	P388D1	MOLT-4	K-562	HT-29
**1**	37.2 ± 4.0	37.8 ± 5.6	― ^*b*^	― ^*b*^
**2**	9.7 ± 1.2	6.0 ± 0.4	5.2 ± 0.8	7.6 ± 2.3
**3**	5.7 ± 1.8	5.3 ± 1.3	12.1 ± 2.4	10.4 ± 2.2
**4**	24.4 ± 4.8	31.2 ± 7.0	21.3 ± 3.7	36.5 ± 7.9
*5-Fluorouracil ^a^*	6.2 ± 0.7	6.9 ± 1.3	33.1 ± 8.9	7.7 ± 0.8

*^a^* Clinical anticancer drug used as a positive control. *^b^* ―: IC_50_ > 40 μM.

**Table 4:** Inhibitory (% Inh) effects of compounds **1**–**4** on superoxide anion generation and elastase release in fMLP/CB-induced human neutrophils at 10 μM.

Compounds	Superoxide Anion			Elastase Release		
	IC_50_ (μM) *^a^*	Inh % ^*b*^		IC_50_ (μM) ^*a*^	Inh % ^*b*^	
**1**	>10	24.8 ± 6.5	*	>10	35.6 ± 1.3	***
**2**	>10	19.4 ± 5.0	*	>10	39.0 ± 2.3	***
**3**	>10	27.7 ± 1.3	***	6.6 ± 1.7	58.8 ± 4.0	***
**4**	8.4 ± 1.1	53.6 ± 1.8	***	6.5 ± 1.1	66.3 ± 6.0	***
**Idelalisib**	0.07 ± 0.01	102.8 ± 2.2	***	0.3 ± 0.1	99.6 ± 4.2	***

*^a^* Concentration necessary for 50% inhibition (IC_50_). *^b^* Percentage of inhibition (Inh%) at 10 μM concentration. Results are presented as mean ± S.E.M. (*n* = 3–4). * *p* < 0.05, *** *p* < 0.001 compared with the control value.

## Supplementary Materials

HRESIMS, ^1^H, and ^13^C spectra of new compounds **1**–**4** are available online at www.mdpi.com/1660-3397/15/9/280/s1. **Figure S1**. HRESIMS spectrum of **1**; **Figure S2**. ^1^H-NMR spectrum (500 MHz) of compound **1** in CDCl_3_; **Figure S3**. ^13^C-NMR spectrum (100 MHz) of compound **1** in CDCl_3_; **Figure S4**. HRESIMS spectrum of **2**; **Figure S5**. ^1^H-NMR spectrum (500 MHz) of compound **2** in CDCl_3_; **Figure S6**. ^13^C-NMR spectrum (125 MHz) of compound **2** in CDCl_3_; **Figure S7**. HRESIMS spectrum of **3**; **Figure S8**. ^1^H-NMR spectrum (400 MHz) of compound **3** in CDCl_3_; **Figure S9**. ^13^C-NMR spectrum (100 MHz) of compound **3** in CDCl_3_; **Figure S10**. HRESIMS spectrum of **4**; **Figure S11**. ^1^H-NMR spectrum (500 MHz) of compound **4** in CDCl_3_; **Figure S12**. ^13^C-NMR spectrum (125 MHz) of compound **4** in CDCl_3_.
