# Supplementary material for: Bioactive Steroids with Methyl Ester Group in the Side Chain from a Reef Soft Coral Sinularia brassica Cultured in a Tank
Source: Mar Drugs. 2017 Sep 1;15(9):280. doi: 10.3390/md15090280 (PMC5618419; doi:10.3390/md15090280)
Supplement: Supplementary file 1 [file marinedrugs-15-00280-s001.pdf]

Bioactive Steroids with Methyl Ester Group in the Side Chain from a Reef Soft Coral *Sinularia brassica* Cultured in a Tank

Chiung-Yao Huang [1], Jui-Hsin Su [2,3], Chih-Chuang Liaw [1], Ping-Jyun Sung [1,2], Pei-Lun Chiang [4], Tsong-Long Hwang [5,6,7], Chang-Feng Dai [8] and Jyh-Horng Sheu [1,9,10,11,*]

[1]Department of Marine Biotechnology and Resources, National Sun Yat-sen University, Kaohsiung 804, Taiwan
[2]National Museum of Marine Biology & Aquarium, Pingtung 944, Taiwan
[3]Institute of Marine Biotechnology, National Dong Hwa University, Pingtung 944, Taiwan
[4]Department of Biochemistry, University of Toronto, Toronto M5G2R3, Canada
[5]Graduate Institute of Natural Products, College of Medicine, Chang Gung University, Taoyuan 333, Taiwan
[6]Research Center for Chinese Herbal Medicine, Research Center for Food and Cosmetic Safety, and Graduate Institute of Health Industry Technology, College of Human Ecology, Chang Gung University of Science and Technology, Taoyuan 333, Taiwan
[7]Department of Anesthesiology, Chang Gung Memorial Hospital, Taoyuan 333, Taiwan
[8] Institute of Oceanography, National Taiwan University, Taipei 112, Taiwan
[9]Department of Medical Research, China Medical University Hospital, China Medical University, Taichung 404, Taiwan
[10]Graduate Institute of Natural Products, Kaohsiung Medical University, Kaohsiung 807, Taiwan
[11]Frontier Center for Ocean Science and Technology, National Sun Yat-sen University, Kaohsiung 804, Taiwan

*To whom correspondence should be addressed. Tel.: 886-7-5252000 ext. 5030, Fax: 886-7-5255020. E-mail: sheu@mail.nsysu.edu.tw.

No	Content	page
**Figure S1**	HRESIMS spectrum of **1**.	**3**
**Figure S2**	^{1}H NMR spectrum (500 MHz) of compound **1** in $CDCl_3$.	**4**
**Figure S3**	^{13}C NMR spectrum (100 MHz) of compound **1** in $CDCl_3$.	**5**
**Figure S4**	HRESIMS spectrum of **2**.	**6**
**Figure S5**	^{1}H NMR spectrum (500 MHz) of compound **2** in $CDCl_3$.	**7**
**Figure S6**	^{13}C NMR spectrum (125 MHz) of compound **2** in $CDCl_3$.	**8**
**Figure S7**	HRESIMS spectrum of **3**.	**9**
**Figure S8**	^{1}H NMR spectrum (400 MHz) of compound **3** in $CDCl_3$.	**10**
**Figure S9**	^{13}C NMR spectrum (100 MHz) of compound **3** in $CDCl_3$.	**11**
**Figure S10**	HRESIMS spectrum of **4**.	**12**
**Figure S11**	^{1}H NMR spectrum (500 MHz) of compound **4** in $CDCl_3$.	**13**
**Figure S12**	^{13}C NMR spectrum (125 MHz) of compound **4** in $CDCl_3$.	**14**

Mass Spectrum SmartFormula Report

Analysis Info

Analysis Name	D:\Data\a1\C1P_000003.d	4/25/2017 3:05:58 PM
Method	broadband first signal	Operator: YU HSIAO-CHING
Sample Name	C1-P	Instrument: BRUKER FT-MS solariX
Comment	ESI Positive	

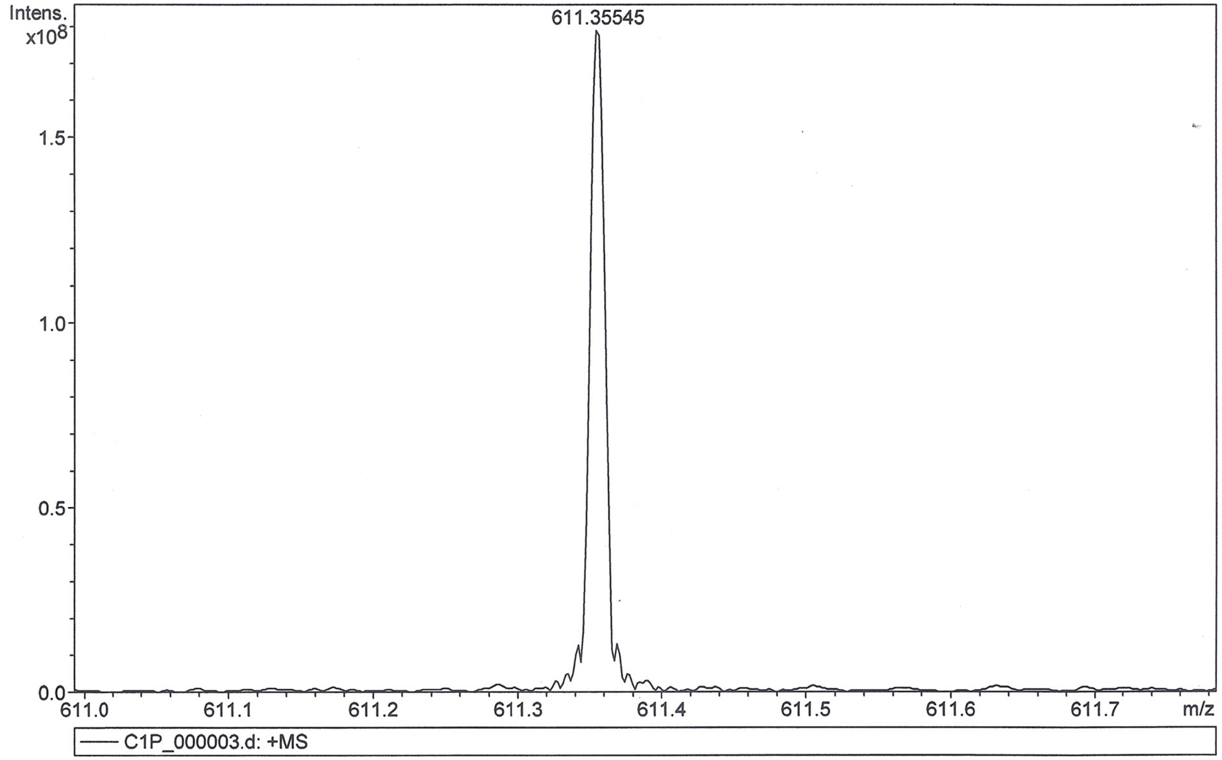

Meas. m/z	#	Formula	Score	m/z	err [mDa]	err [ppm]	mSigma	rdb	e⁻ Conf	N-Rule
611.35545	1	C 34 H 52 Na O 8	100.00	611.35544	-0.02	-0.02	15.5	8.5	even	ok

Figure S1. HRESIMS spectrum of **1**.

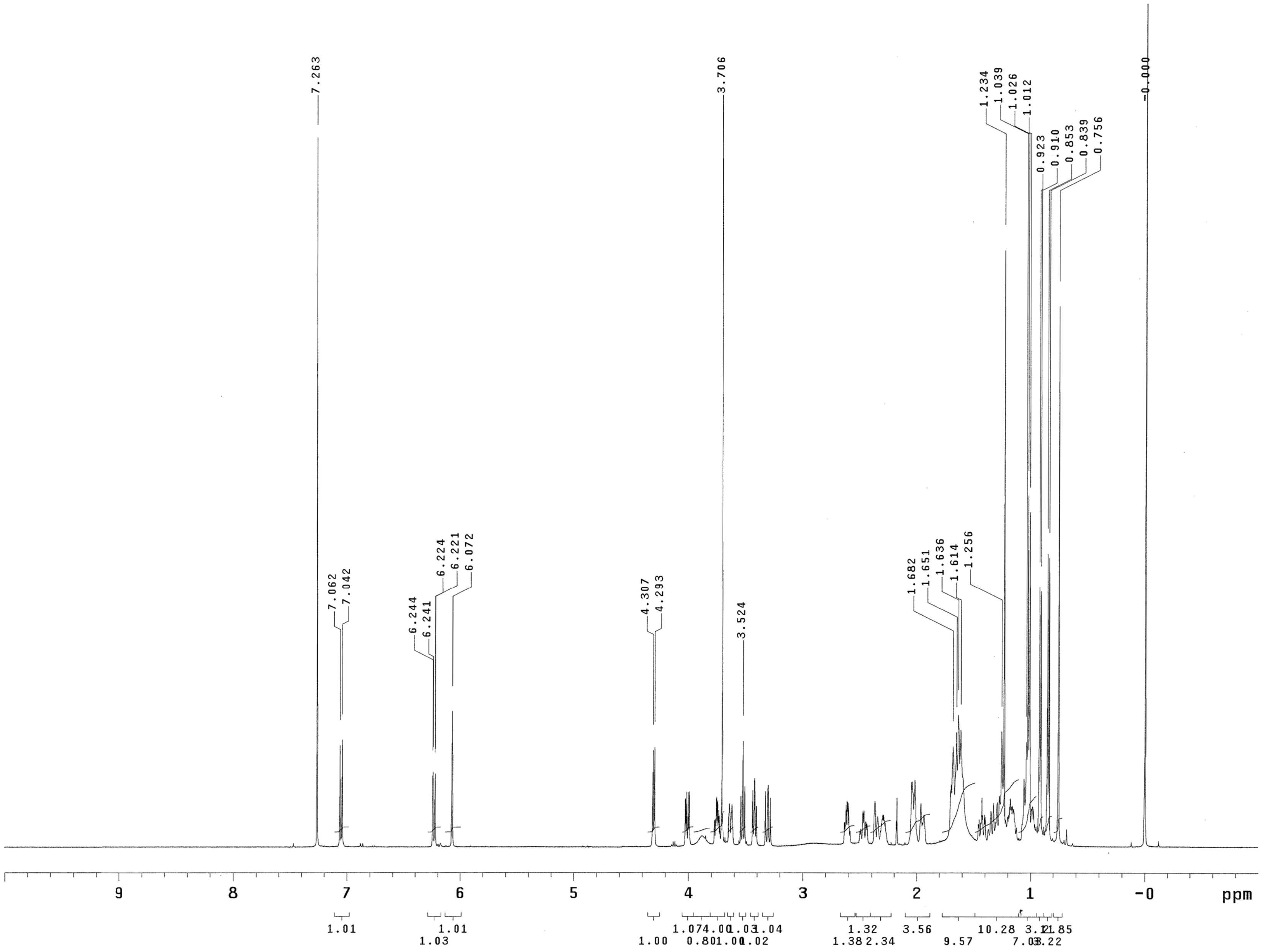

Figure S2. ^{1}H NMR spectrum (500 MHz) of compound **1** in $CDCl_3$.

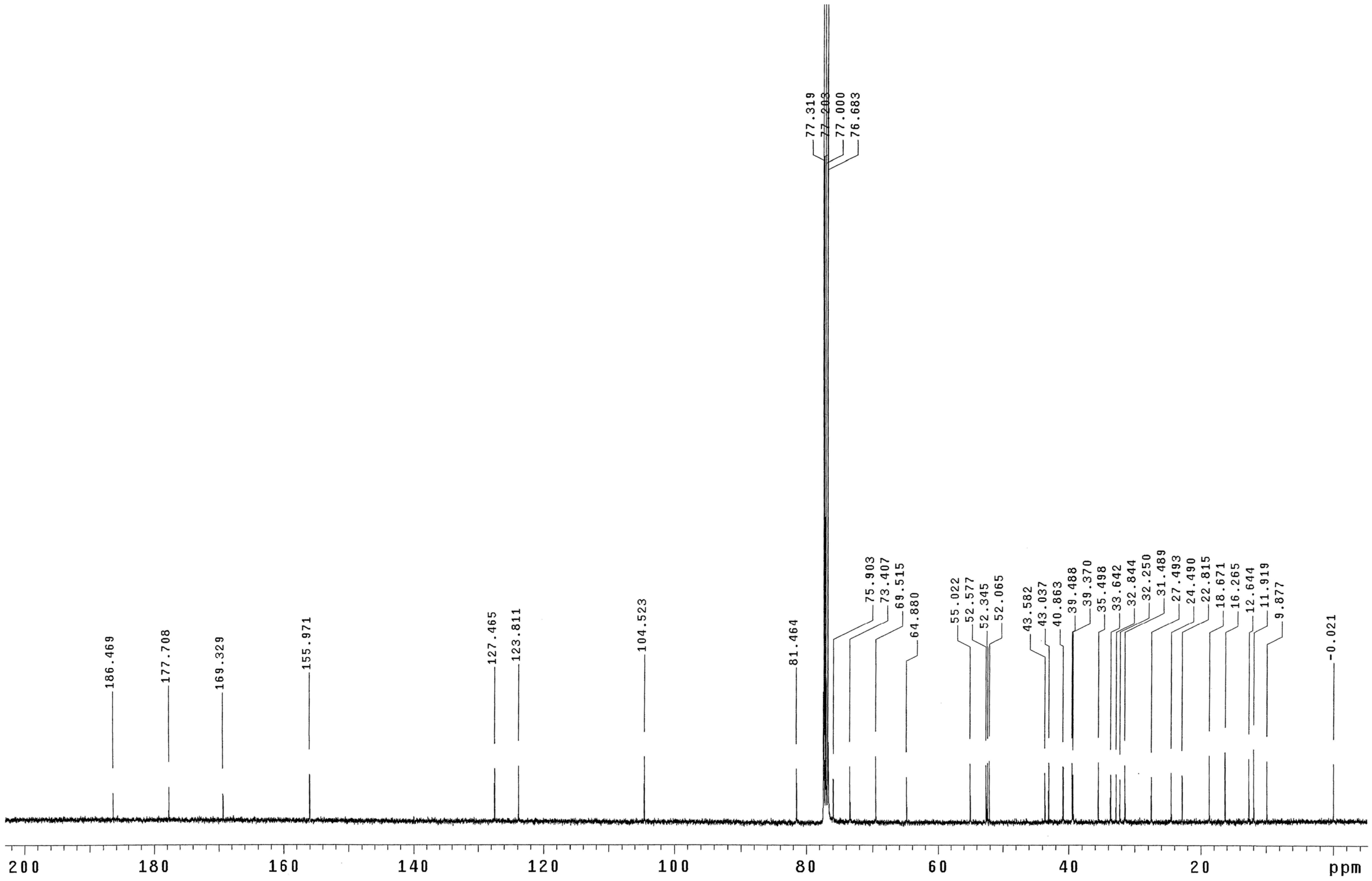

Figure S3. ^{13}C NMR spectrum (100 MHz) of compound **1** in $CDCl_3$.

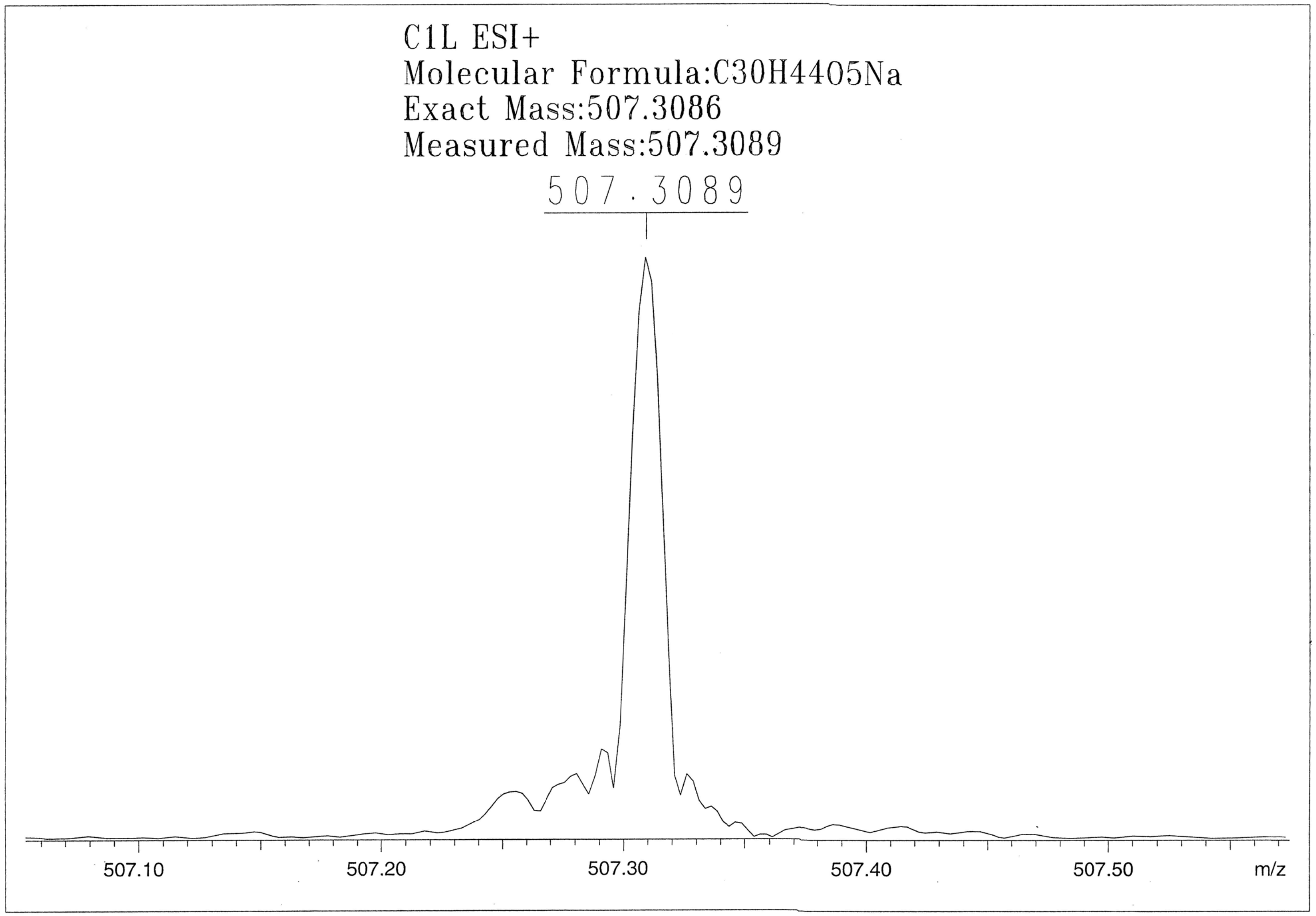

Figure S4. HRESIMS spectrum of **2**.

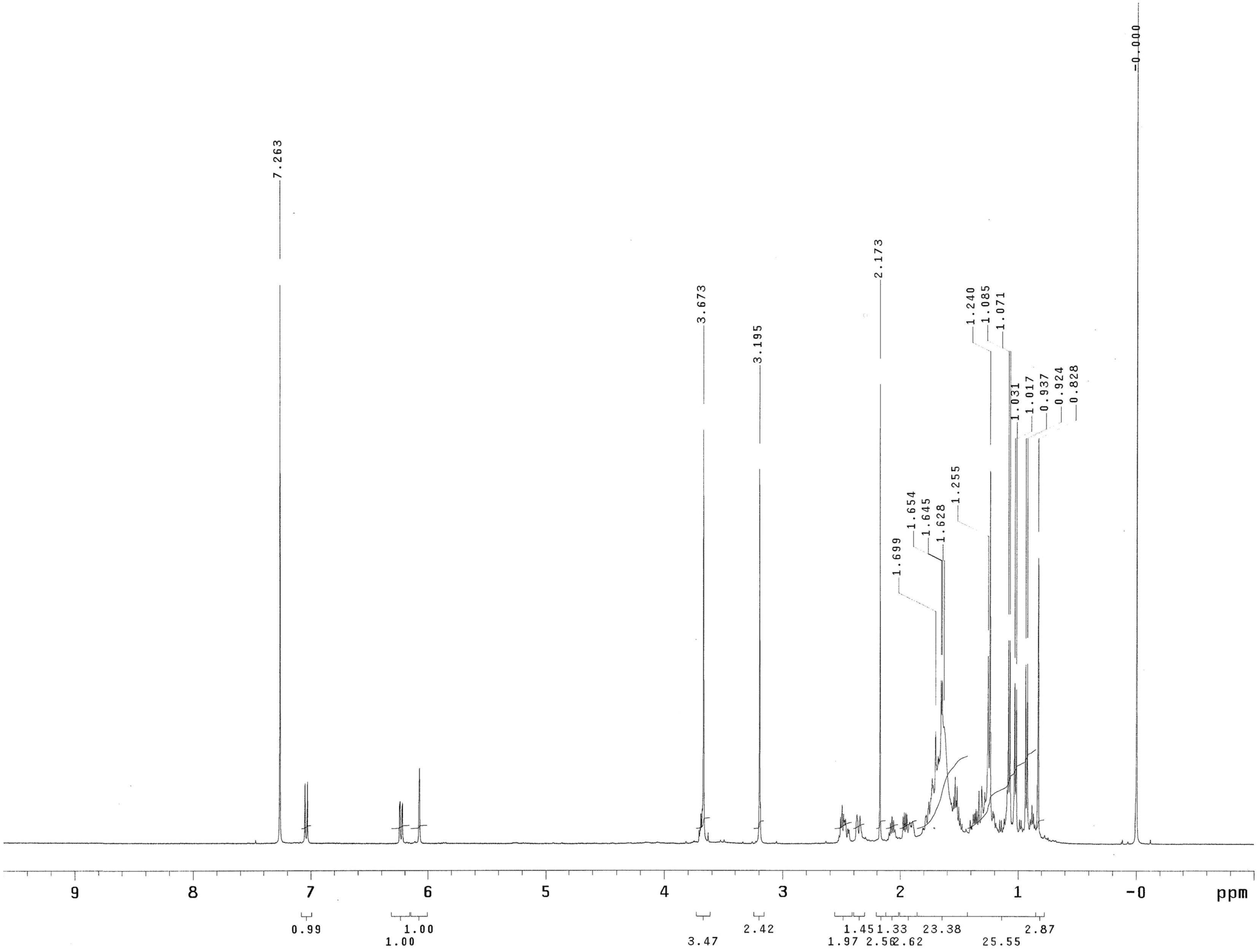

Figure S5. ^{1}H NMR spectrum (500 MHz) of compound **2** in $CDCl_3$.

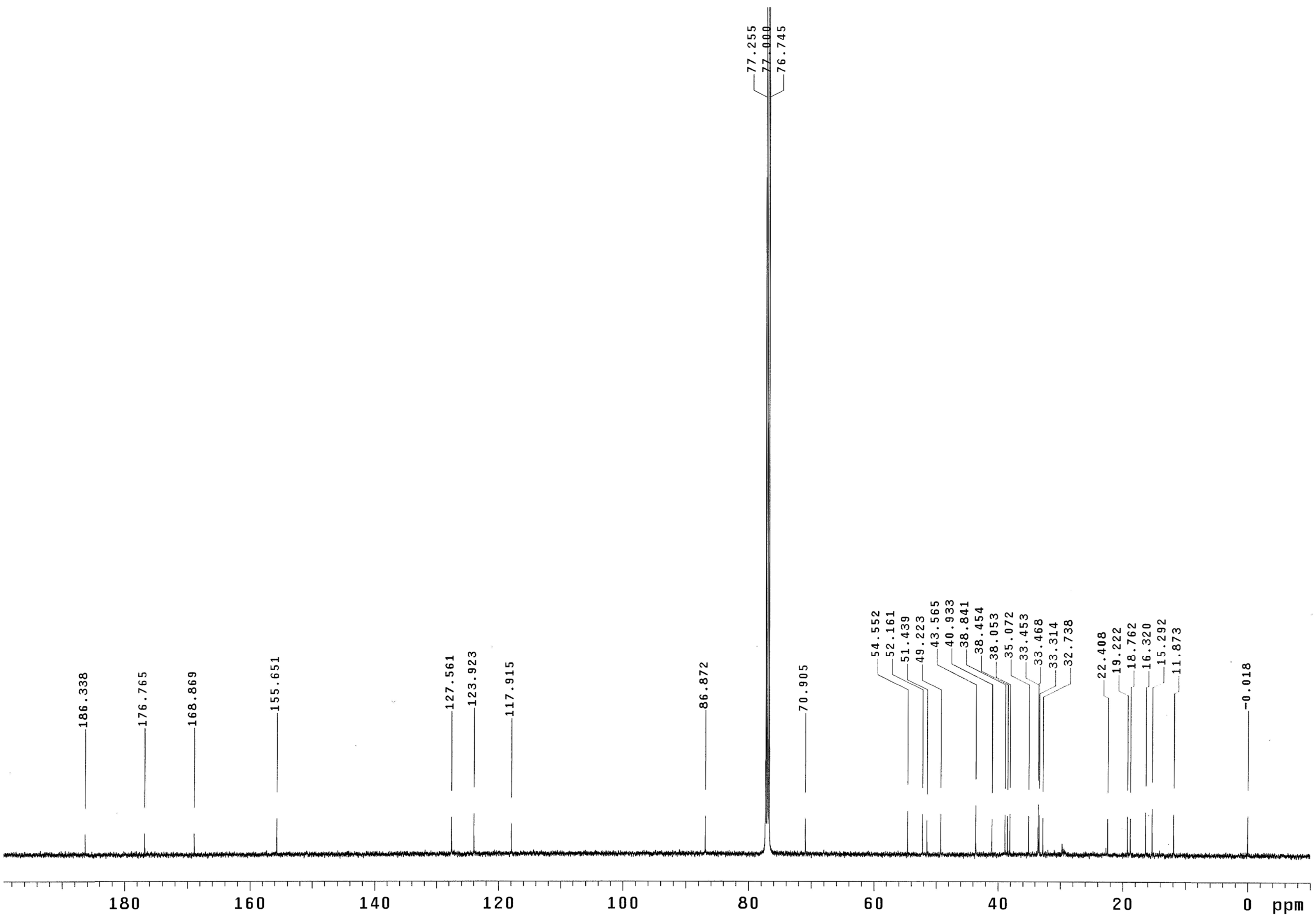

Figure S6. ^{13}C NMR spectrum (125 MHz) of compound **2** in $CDCl_3$.

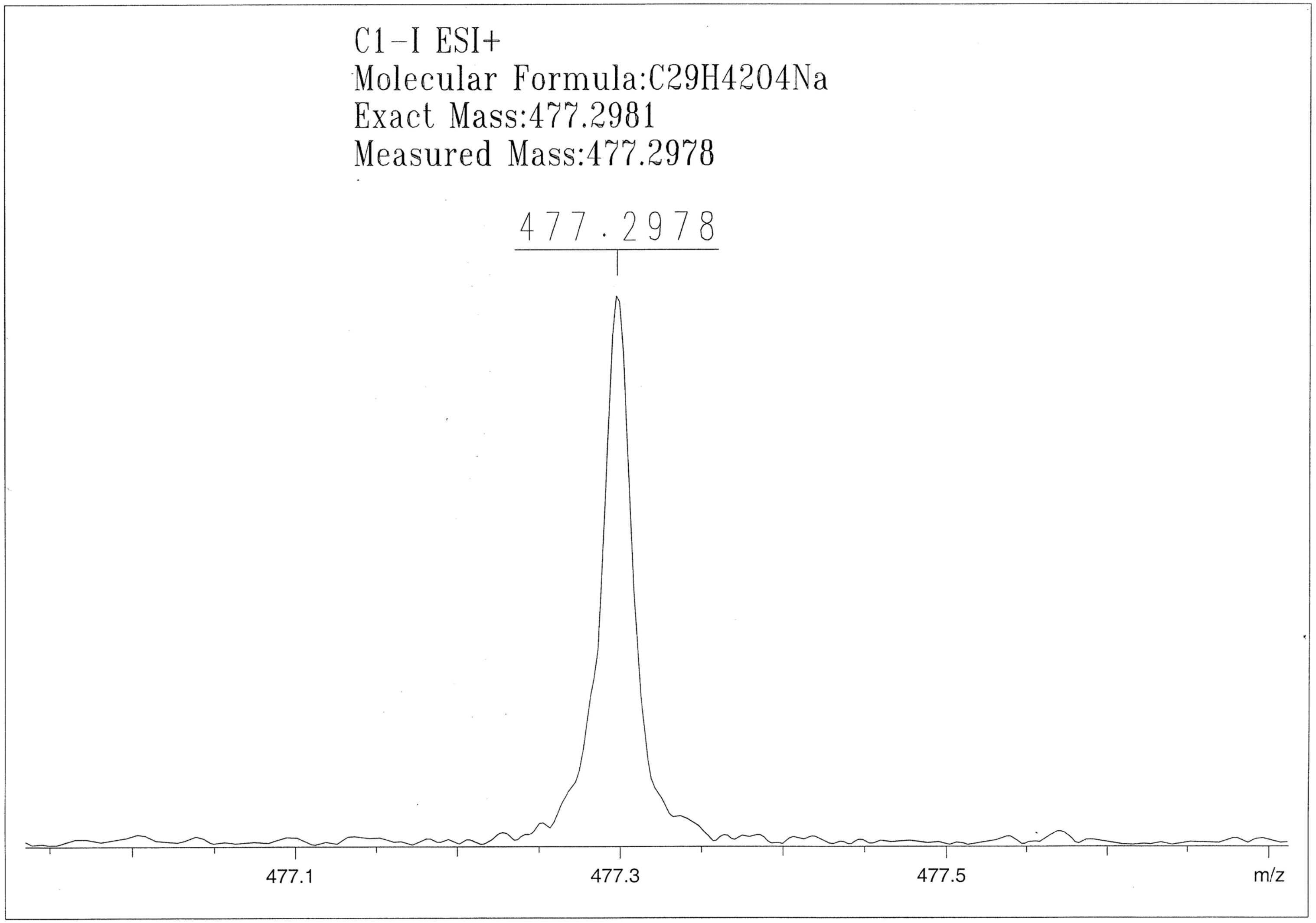

Figure S7. HRESIMS spectrum of **3**.

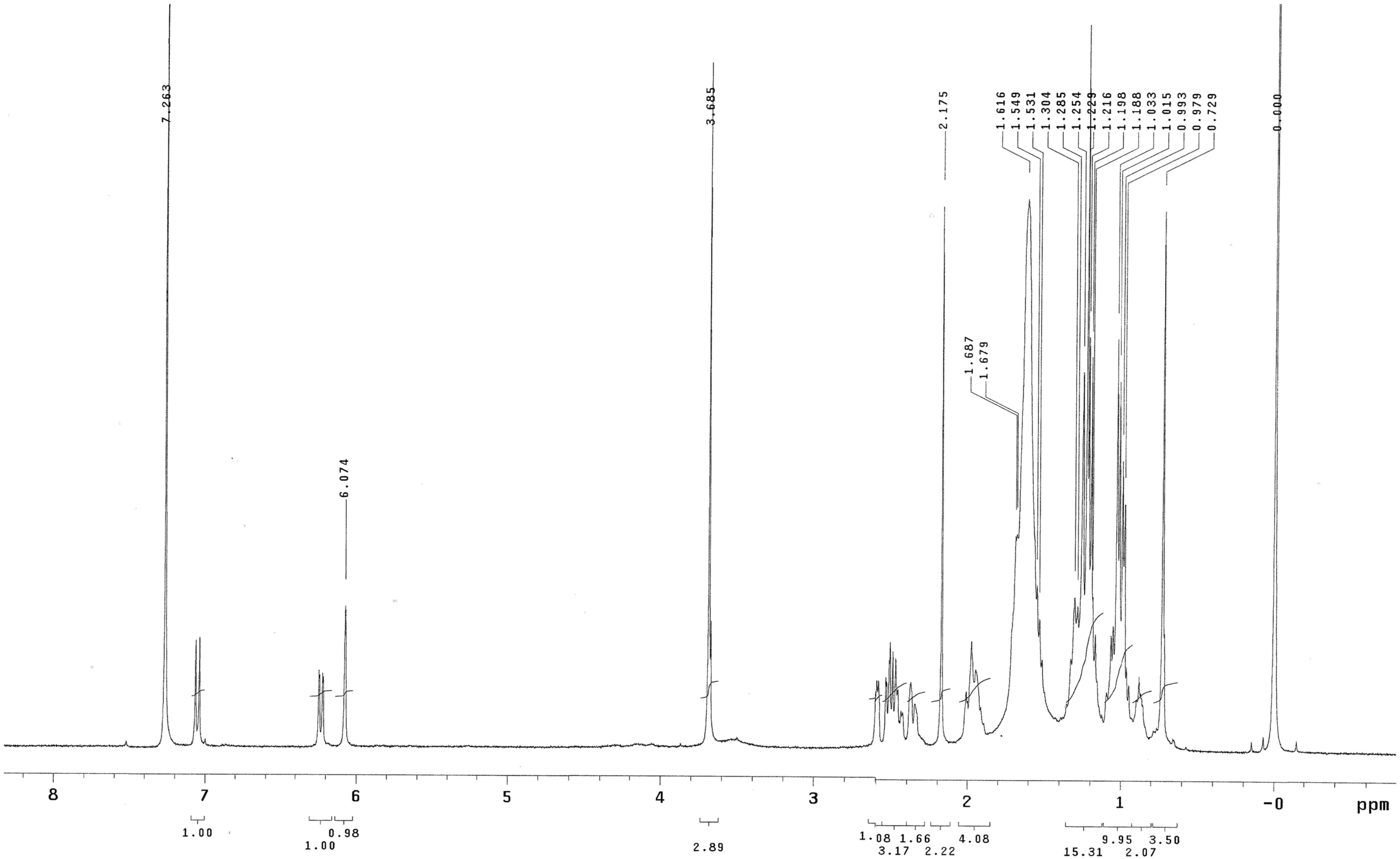

Figure S8. ^{1}H NMR spectrum (400 MHz) of compound **3** in $CDCl_3$.

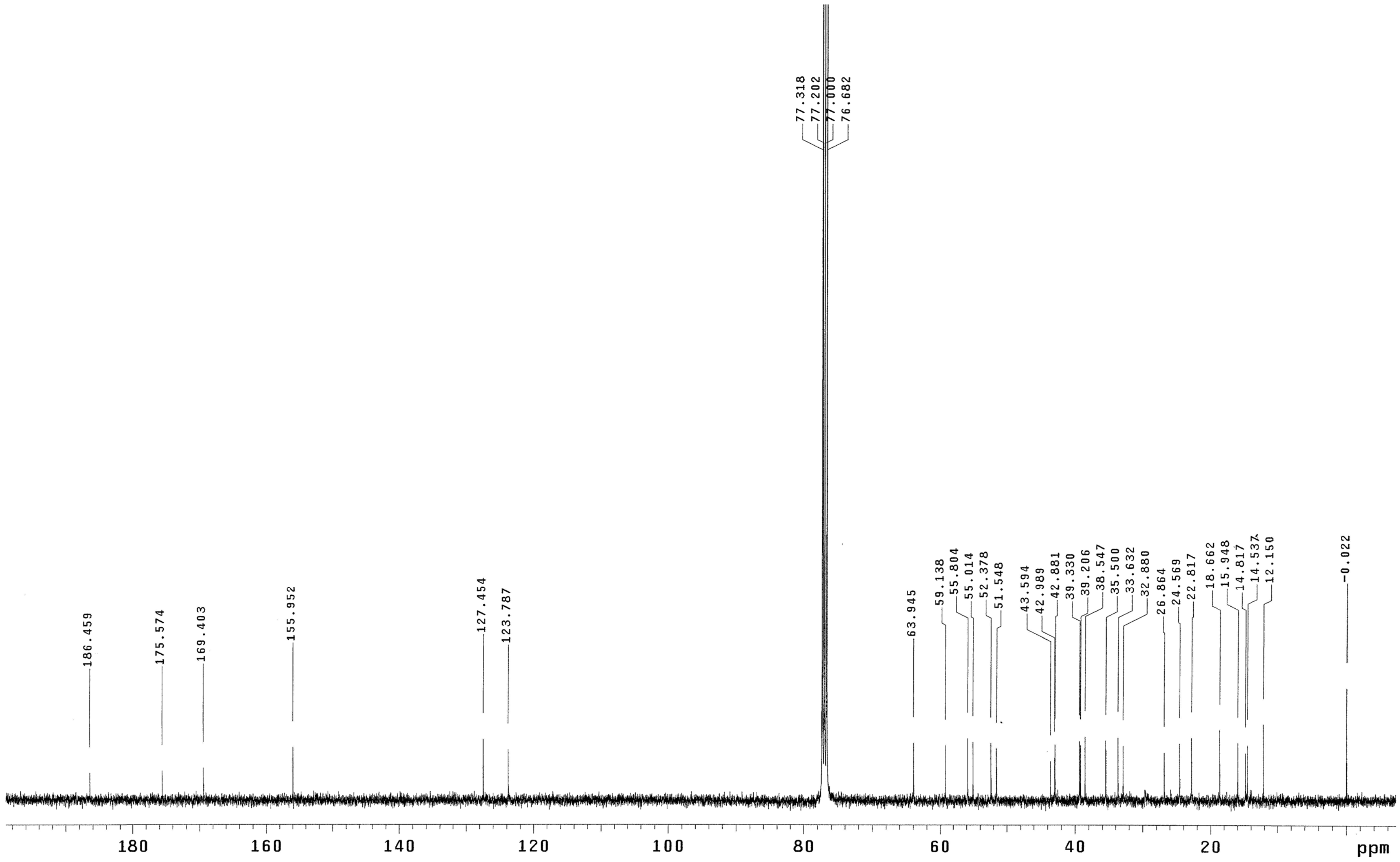

Figure S9 ^{13}C NMR spectrum (100 MHz) of compound **3** in $CDCl_3$.

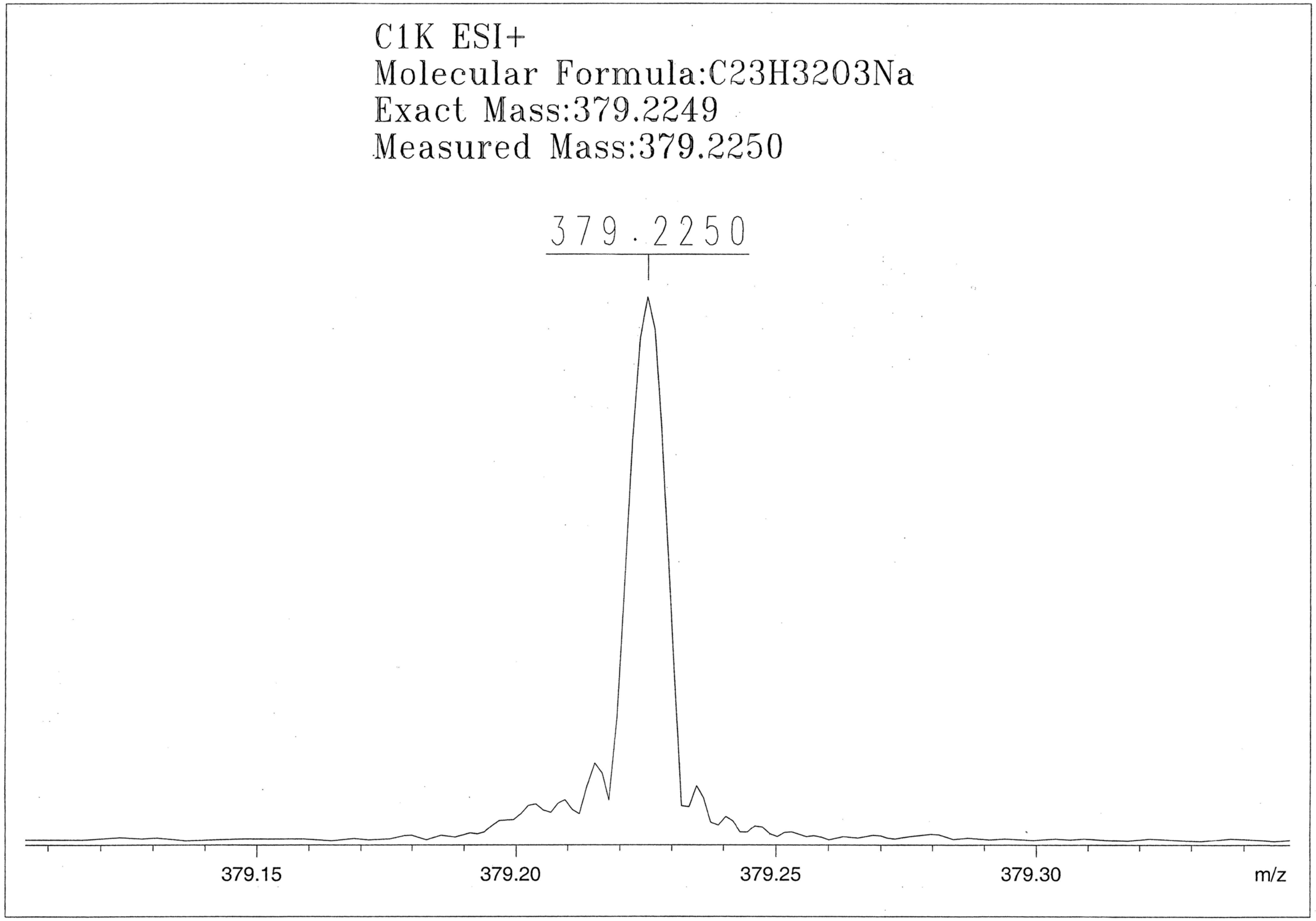

Figure S10. HRESIMS spectrum of **4**.

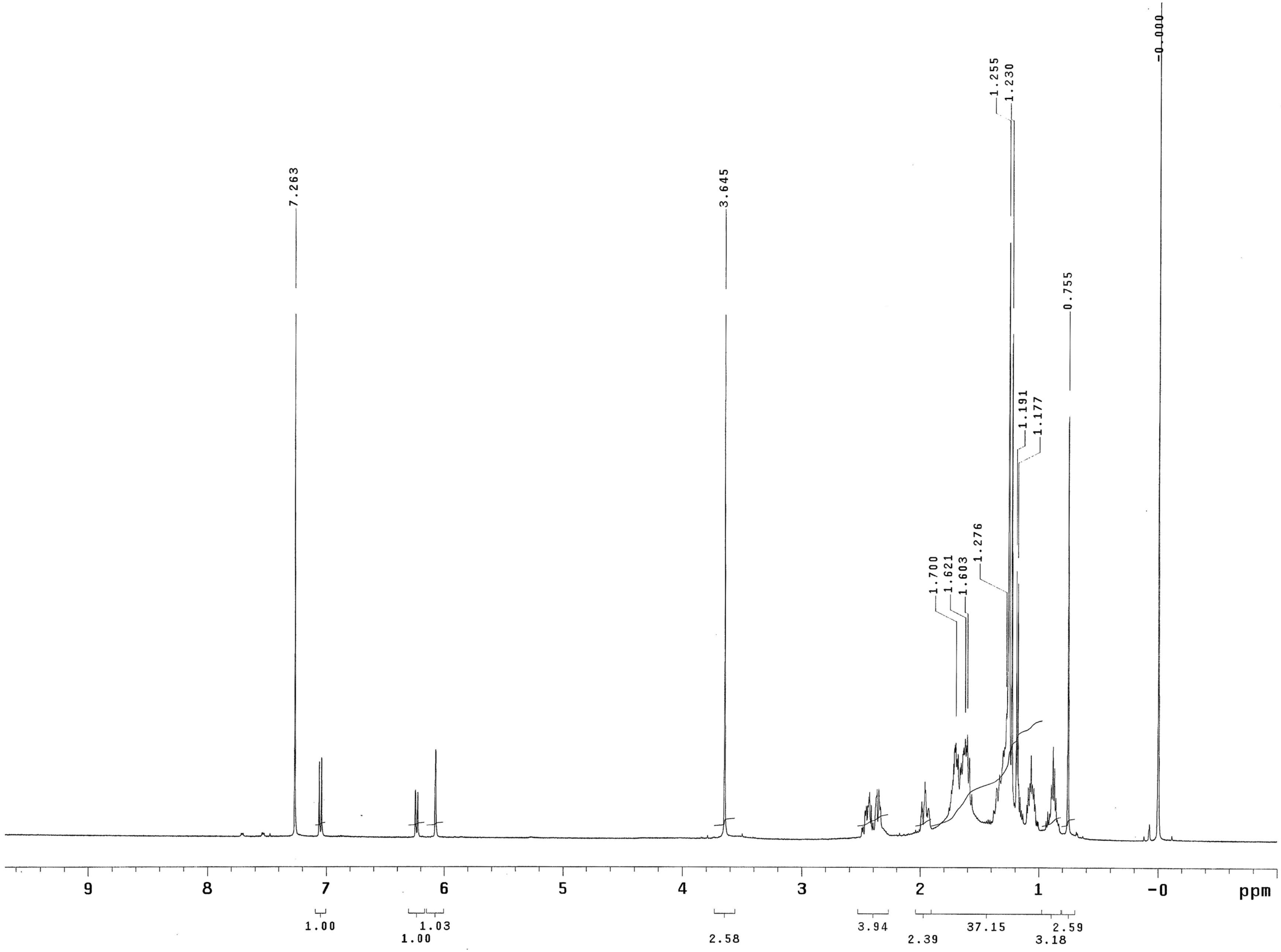

Figure S11. ^{1}H NMR spectrum (500 MHz) of compound **4** in $CDCl_3$.

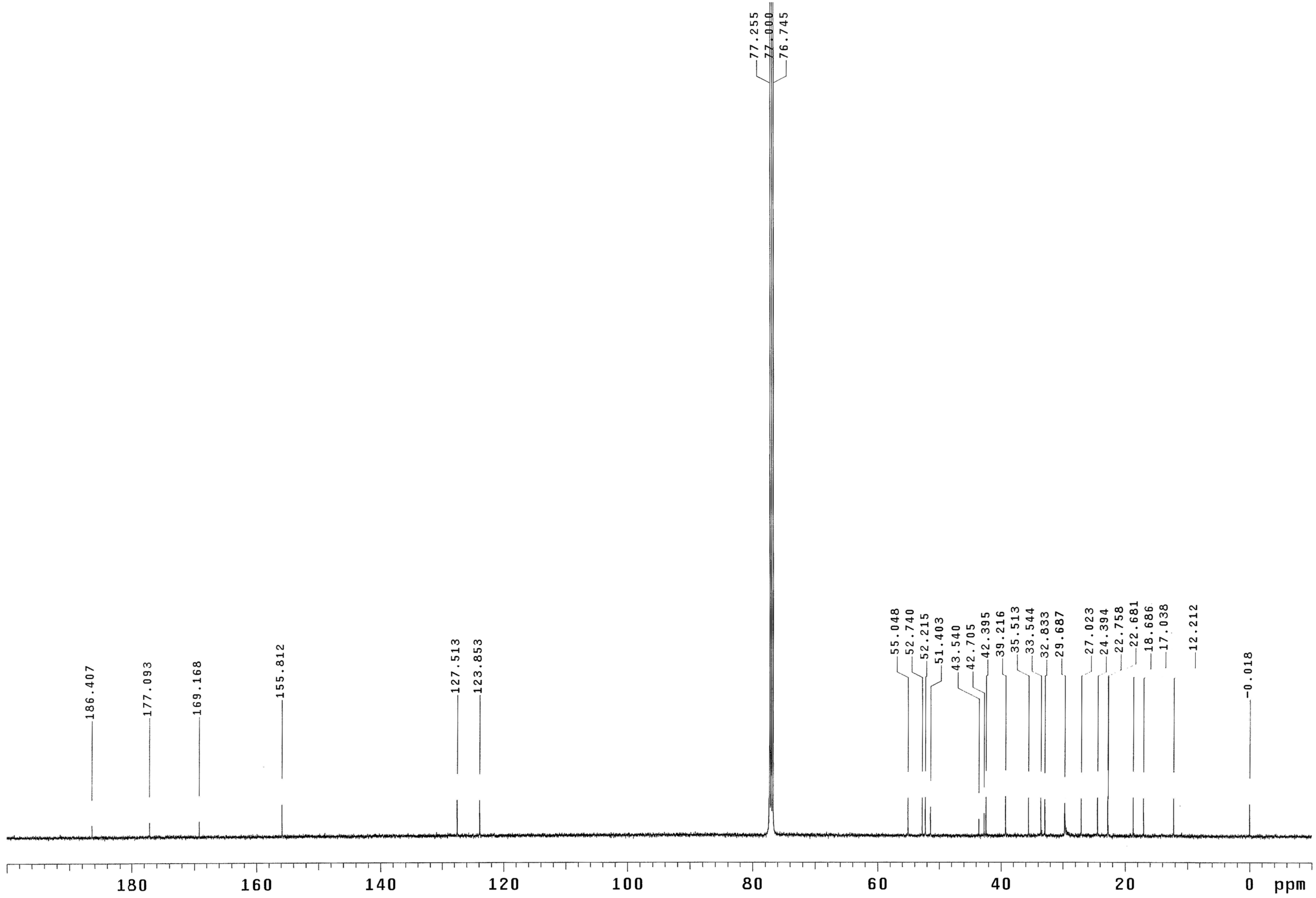

Figure S12. ^{13}C NMR spectrum (125 MHz) of compound **4** in $CDCl_3$.
